# Holder Pasteurization Affects the VOCs and Lipid Profile of Human Milk

**DOI:** 10.3390/foods15071118

**Published:** 2026-03-24

**Authors:** Cristiane Mori, Christopher Pillidge, Harsharn Gill

**Affiliations:** School of Science, RMIT University, Melbourne, VIC 3083, Australia; cristiane.mori@rmit.edu.au (C.M.); christopher.pillidge@rmit.edu.au (C.P.)

**Keywords:** human milk banks, donor human milk (DHM), lipids, volatile organic compounds (VOCs), FT-IR, GC-MS, infant nutrition

## Abstract

Donor human milk (DHM) provided by human milk banks is considered the optimal feeding alternative to mother’s own milk for premature or medically compromised infants. Before distribution, DHM is subjected to Holder pasteurization (HoP) by milk banks to eliminate potential pathogens. In this study, FT-IR, GC and GC-MS were applied to characterize changes in the volatile organic compounds (VOCs) and lipid components of human milk (HM) samples that were treated by HoP. FT-IR analysis revealed changes in specific band regions, indicating modifications to triglycerides and fatty acid (FA) organization and possible disruption of the milk fat globule membrane. There was also an increase in ester groups, suggesting that HoP increases lipid oxidation. GC analysis showed a reduction in long-chain FAs, including certain omega-3 and omega-6 polyunsaturated FAs (PUFAs). GC-MS analysis showed that HoP-treated samples contained higher levels of alkanes, aldehydes, aromatics and ketones than raw HM. Conversely, other compounds, including furans, and alkynes, were found exclusively in pasteurized HM. These results show that HoP affects the lipid and VOC components of HM, highlighting the need for research into alternative pathogen elimination strategies in human milk bank practices.

## 1. Introduction

Human milk (HM) is a nutritionally complex food, with lipids representing approximately 3–5% (*w*/*v*) of its composition. These lipids, predominantly triglycerides, provide nearly half of the total energy intake for breastfed infants during the first six months of life, with fully breastfed infants ingesting an estimated 30 g of lipids per day [1,2,3]. Such lipids are essential for infant growth, supplying critical fatty acids (FAs) that support brain development, metabolic regulation, and hormonal function [4,5]. The FA profile of HM is different to that of infant formula (IF), highly variable between mothers, and can be influenced by gestational age at birth, maternal health, dietary intake and metabolic status [6,7]. Maternal dietary patterns, particularly the intake of mono- and polyunsaturated FAs, and lactation stage further shape the composition of milk lipids [8,9,10].

Beyond such biological variation, the handling and processing of HM, especially donor human milk (DHM), may significantly alter its lipid integrity. Pasteurization, particularly Holder pasteurization (HoP) practiced by milk banks worldwide, is the internationally recommended method for ensuring microbiological safety but may also modify lipid structure, fatty acid profiles, and the entire macronutrient matrix [11]. Storage conditions can also disrupt the milk fat globule membrane and promote lipolysis, with free fatty acids (FFAs) detectable within hours at room temperature and even in samples stored at −20 °C [12]. Freezing and prolonged storage may also induce lipid peroxidation, generating bioactive oxidation products such as oxylipins [13]. While various analytical methods have been applied to investigate milk lipids, including gravimetric assays, a spectrometric analysis of esterified FAs, infrared-based milk analyzers and traditional gas chromatography (GC) and liquid chromatography (LC) methods [2,14,15], the extent to which HoP affects HM lipids remains insufficiently characterized.

The objective of this study was to evaluate how HoP affects the volatile organic compounds (VOCs) and the molecular structure of lipids in DHM by analyzing changes in key functional groups using Fourier-transform infrared (FT-IR) spectroscopy, coupled with GC methods. The use of FT-IR and other forms of IR spectroscopy represent a useful approach to this question [16,17,18,19]. By examining spectral regions associated with FAs, triglyceride configuration, and lipid–protein interactions, we aimed to assess the structural modifications in the DHM lipid matrix following thermal processing by HoP.

## 2. Materials and Methods

### 2.1. Human Milk Samples and Ethics Approval

This study was approved by the RMIT University Human Research Ethics Advisory Committee (Reference 22831). Ten human milk (HM) samples donated by Australian Red Cross Lifeblood were used for analyses. Selection criteria for donors, milk collection protocols, milk processing, handling and storage were based on the standards set down by the Australian Government Department of Health and Aged Care. After receival, each HM sample (approximately 100–200 mL) was thawed at 4 °C, then homogenized and divided into two batches: one batch was kept unpasteurized (raw), and the other was pasteurized under Holder pasteurization (HoP) conditions (62.5 °C for 30 min in a water bath). During pasteurization in sealed tubes, the internal temperature of the milk was continuously monitored using a reference tube. Samples were considered under pasteurization once the milk reached 62.5 °C, after which this temperature was maintained for 30 min before rapid cooling to 4 °C. Samples were aliquoted in 2 mL glass tubes with Teflon-lined screw caps (Thermo Fisher Scientific, Scoresby VIC 3179, Australia) for FT-IR and GC-FID analyses or 20 mL head-space vials (Agilent Technologies, Mulgrave, VIC 3170, Australia) for volatiles analysis by SPME and GC-MS, and stored at −80 °C until analyses.

### 2.2. FT-IR Analysis

A PerkinElmer FT-IR spectrometer (Spectrum two, PerkinElmer, Glen Waverley, VIC 3150, Australia) was used to collect the Attenuated Total Reflection Mid-Infrared (ATR MIR) spectra of the HM samples. A sample drop (200 µL) of raw and pasteurized HM samples was placed on the spectrometer window. IR spectra were recorded in duplicate for each sample at room temperature at a resolution of 4 cm^−1^ in the mid-infrared wavenumber range between 400 and 4000 cm^−1^ (average 16 scans), and water was used as the background. The window was cleaned with ethanol between samples to avoid cross-contamination. After each measurement, a new background spectrum was collected.

### 2.3. GC-FID Analysis

To explore changes in the lipid fraction, FAs were extracted from HM samples and converted to methyl esters for analysis with gas chromatography coupled with flame ionization detection (GC-FID), as described by Cruz-Hernandez [14]. This process, involving methanol and an acid catalyst, transforms FFAs and triglycerides (small-, medium- and long-chain FAs) within the same reaction conditions. A 300 µL volume of raw and pasteurized HM was added to glass test tubes with Teflon-lined screw caps (Corning, NY 14831, USA), followed by 300 µL of a methyl undecanoate 3.1 µg/mL as an internal standard solution (Merck, Bayswater VIC 3153, Australia). Subsequently, 2 mL of methanol, 2 mL of methanolic HCl (3N, Merck) and 1 mL of n-hexane were added. Test tubes were shaken vigorously and heated at 100 °C for 60 min, with occasional additional shaking. After cooling the samples to room temperature, 2 mL of water was added, followed by vigorous shaking. Test tubes were then centrifuged at 1200× *g* for 5 min and the upper phase (n-hexane) was transferred to vials for GC injection. Internal standard methyl undecanoate and 37-component FAME mix were purchased from Merck. Methanolic HCl (3N) for GC derivatization, methanol and n-hexane were purchased from Merck.

Methylated FAs were injected into a gas chromatography coupled with a flame ionization detector (GC-FID. HP/AGILENT, Model 6890B, Santa Clara, CA 95051, USA). A fused silica capillary column (100 m × 0.25 mm × 0.2 µm film thickness, Supelco, Merck) was used for fatty acid methyl ester (FAME) analysis. A 37-component FAME mix (Merck) was used as a standard for the identification and quantification of compounds. Helium was used as the carrier gas at an injection rate of 2.1 mL/min. A total of 1 µL of each sample was injected in split mode at a 5:1 ratio with initial oven temperature set at 140 °C for 5 min and increasing until 250 °C at a rate of 5 °C/min with an equilibration time of 0.5 min.

### 2.4. GC-MS Analysis

Glass headspace vials (as described previously) with screw caps were used for the headspace extraction of volatile organic compounds (VOCs). A total of 1 mL of DHM, raw or pasteurized, was added individually to headspace vials. DHM VOCs were extracted with a solid-phase microextraction (SPME) fiber (DVB/Carbon-WR/PDMS/10, Dark Gray, Agilent, Santa Clara, CA 95051, USA) and analyzed through gas chromatography coupled with mass spectrometry (GTC-MS SQ Agilent 8890/5977). An empty headspace vial was used as blank to discard contaminants from Teflon and SPME fiber. For VOC extraction, the headspace vials containing samples were equilibrated (heating at 50 °C for 15 min with occasional shaking at 250 rpm), and the extraction process using SPME fiber was conducted for a duration of 5 min. The fiber was then desorbed in the gas chromatography inlet at 120 °C for 0.2 min, and helium was used as the carrier gas in splitless mode at a constant flow rate of 3 mL/min. The separation was achieved through a 19091N-133I-HP-INNOWax (30 m × 0.25 mm × 0.25 µm) column. Oven temperature conditions were set at an initial temperature of 40 °C for 1 min, then increased at a 10 °C/min rate until 250 °C and held for 5 min. The resulting chromatograms were exported to MassHunter software.

### 2.5. Statistical Analyses

FT-IR spectra were imported to PeakSpectroscopy Software (v. 3.00) for peak annotation and peak picking. Statistical analysis was conducted in R Studio (v. 4.3.2) with ChemoSpec package (v. 6.1.10). Raw data were imported and visually inspected for quality control, followed by baseline correction. Then, replicates were aligned and processed for noise deletion, peak binning, and data cleaning. Exploratory data analysis was conducted in R Studio.

For the identification and quantification of FAs, raw GC-FID spectra were imported to MassHunter Qualitative Analysis software (v.10.0, Agilent Technologies, Santa Clara, CA 95051, USA). Identified peaks were quantified compared to the FAME-Mix standard curve, and the concentration of each FA was reported as group average ± standard error of the mean. The intensity of the identified peaks was normalized using the autoscaling method, where values were mean-centered and divided by the standard deviation [20]. A one-way ANOVA with Tukey post hoc comparison test was conducted using GraphPad Prism (v 10.1.2) to determine the statistical significance of different concentrations of equivalent FAs between raw and pasteurized groups. Multivariate statistical analysis was performed using R Studio and MetaboAnalystR (v. 4.0) package. Principal component analysis (PCA) was used to group samples based on the effect of individual variances and heat-treatment on the FA profile. Unsupervised clustering analysis was used to determine sample grouping based on the arrangement of the treatment groups (P and R) and individuals based on their similarities.

VOCs peak annotation and library search were conducted in MassHunter Qualitative Analysis software using the National Institute of Standards and Technology (NIST) for the identification of deconvoluted peaks. Massbank spectral library was used for spectra identification. Chemical compounds were assigned to peaks with a minimum mass spectral similarity of 80%. The databases MetaCyc, Chemical Entities of Biological Interest (ChEBI), LOTUS, and the human metabolome database were used to determine the likely origins and pathways of identified VOCs, as well as possible physiological effects [21,22,23,24]. The Norman database was consulted for migrating and extractable food contact chemicals involved in food packaging [25]. A list containing identified compounds and their intensity was analyzed with MetaboAnalystR. Raw data was normalized through autoscaling, i.e., mean-centered and divided by the standard deviation. PCA and unsupervised clustering were used to determine the effect of individual variations and heat-treatment (raw vs. pasteurized) in milk’s VOC composition.

## 3. Results

### 3.1. FTIR Analysis

**Figure 1:**
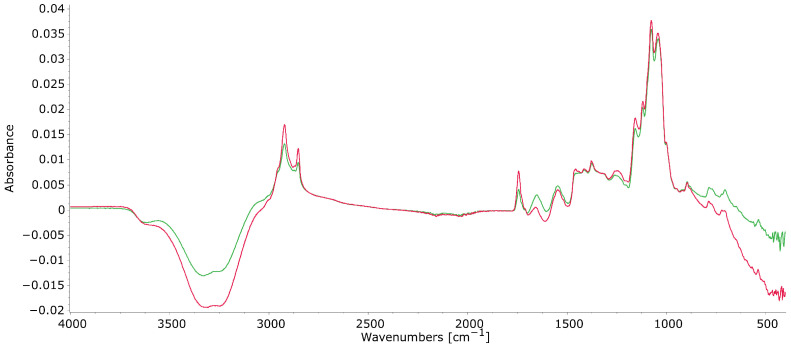
First derivative of the FT-IR spectra of raw (green) and pasteurized (red) human milk samples, illustrating the absorbance as a function of wavenumbers (cm^−1^) across the range 4000–500 cm^−1^.

Figure 1 shows the FT-IR analysis spectra of raw (green) and pasteurized (red) HM samples. The complete spectrum includes wavebands representing functional groups from major HM components, such as lipids, proteins, and carbohydrates, as well as functional groups resulting from the interactions between these components.

**Figure 2:**
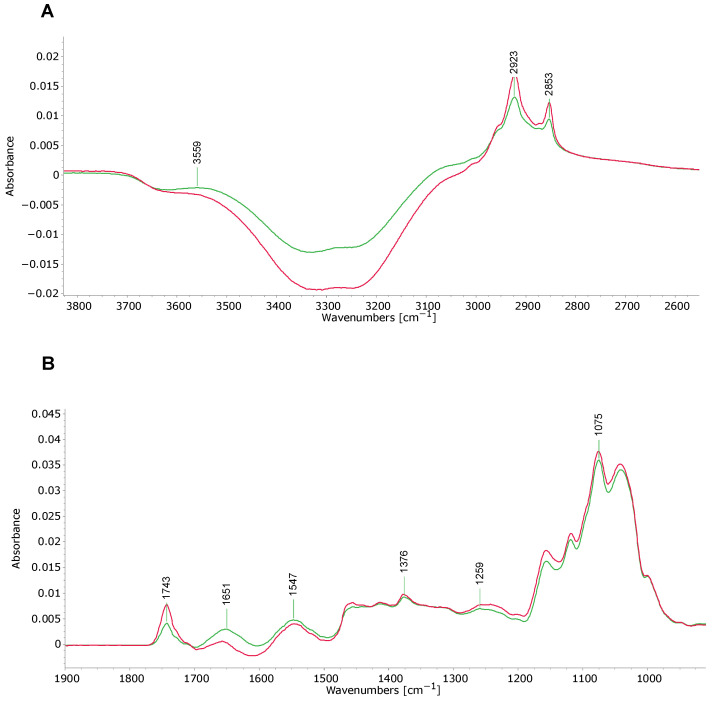
FT-IR spectra of human milk in the range of 3800–2600 cm^−1^ (**A**) and 1900–900 cm^−1^ (**B**) for raw (green) and pasteurized (red) HM samples. Wavenumbers (cm^−1^) indicative of specific functional groups are indicated.

Enlarging specific regions shows these differences more clearly. Figure 2 shows spectra at wavelength ranging from 3800 to 2600 (A) and 1900–900 cm^−1^ (B), respectively. Peaks at wavenumbers 2923 cm^−1^ and 2853 cm^−1^ correspond to the aliphatic methylene (CH_2_) functional group associated with lipid–protein interactions (Figure 2A). At both wavelengths, the pasteurized samples exhibited higher absorbance (group averages 0.0167 and 0.0121, respectively) compared to the raw milk samples (0.0133 and 0.0095, respectively). However, no differences between groups were observed at the wavelength 2959 cm^−1^, which corresponds to the methyl functional group of protein–lipid interactions. The peak at wavenumber 1743 cm^−1^ (Figure 2B) corresponds to the carbonyl (C=O) ester groups of lipids and to lipid–protein interactions containing C=O. At this wavelength, the pasteurized HM samples showed higher absorbance (0.0076) compared to raw HM samples (0.0042). These findings indicate that HoP alters functional groups in HM that are related to lipid–protein interactions.

The Amide I protein functional group peak was observed at 1651 cm^−1^ (Figure 2B), corresponding to C=O stretching, nitrile (C≡N) stretching, and primary amine (N-H) bending vibrations [17]. The Amide I band represents interactions of proteins with lipids and water. In this band, the raw milk sample produced a higher absorbance (group average 0.0027) compared to the pasteurized milk samples (0.0005). Similarly, an Amide II protein group peak was detected at wavelength 1547 cm^−1^, with raw milk samples showing slightly higher absorbance (0.0046) compared to the pasteurized milk samples (0.0041). This functional group is related to protein interactions with lipids. An Amide III peptide bond vibration was detected at wavelength 1259 cm^−1^ (Figure 2B) corresponding to complex displacements in the milk mixture associated with CN, NH, CH, and alkene (C≡C) vibrations. In this region, the pasteurized milk samples exhibited higher absorbance (0.0075) compared to raw milk samples (0.0071), suggesting that HoP reduces protein–lipid interactions. Overall, these results clearly show that pasteurization significantly affects protein interactions with other components in HM.

**Figure 3:**
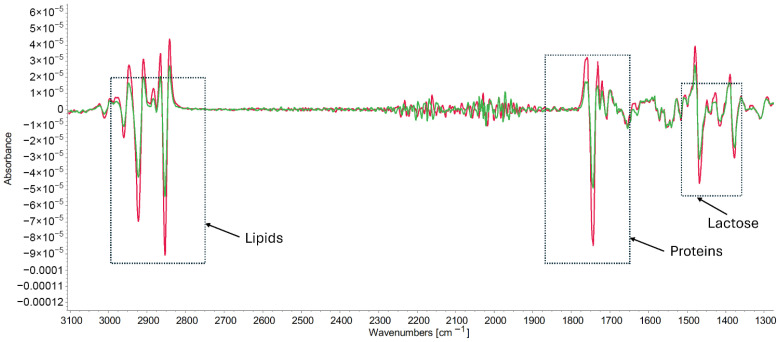
Second derivative of FT-IR spectra of raw (green) and pasteurized (red) human milk samples.

To resolve the extensive overlapping of absorption peaks in the spectra, a second derivative mathematical approach was applied. The second derivative of the combined data revealed some peak differences that were not evident in the original FT-IR spectra (Figure 3), including differences in peak intensity appearing around 2959 cm^−1^, representing CH_3_ functional groups of lipid–protein interactions, and at 1467 cm^−1^, related to hydroxyl (OH) and CH_2_ interactions between lipids and lactose. These findings indicate that using the second derivative technique can uncover subtle differences in the spectral data, highlighting that HoP primarily impacts lipid and protein interactions in HM.

### 3.2. Gas Chromatography Analysis

#### 3.2.1. Fatty Acid Composition of HM by GC-FID

**Figure 4:**
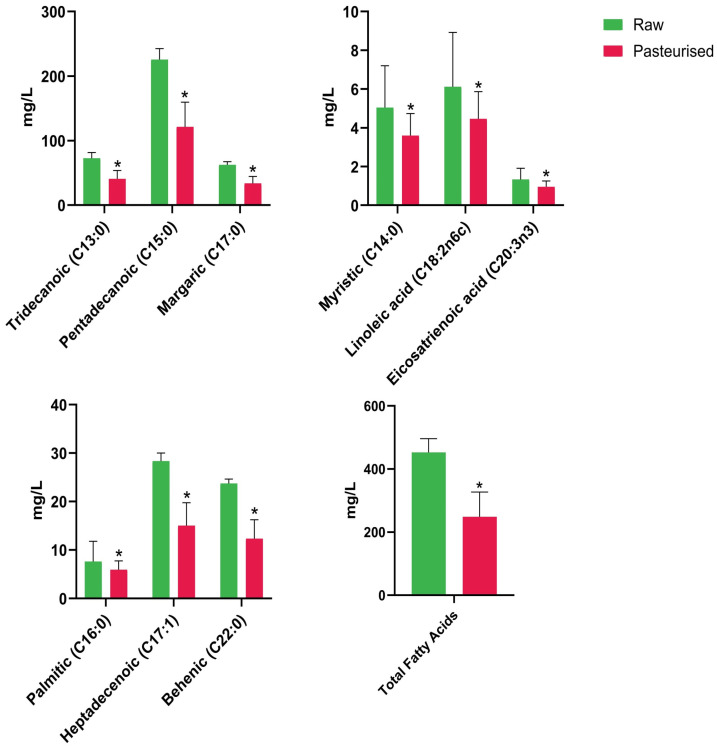
Fatty acid (FA) concentrations following pasteurization; results are shown as average ± SEM. A * indicates statistically significantly different values (raw vs. pasteurized; *p* ≤ 0.05). Only FAs detected at significant levels in all HM samples are shown.

The concentrations of fatty acids (FAs) detected using this method are shown in Figure 4, with data pooled by group (raw vs. pasteurized). The method and conditions used primarily targeted medium- and long-chain FAs (13 carbons or more), not short-chain FAs (SCFAs), many of which were presumably lost. The GC-FID chromatograms are shown in Supplementary Figure S1. Important FAs detected included the monounsaturated FA heptadecenoic acid (C17:1) and three polyunsaturated FAs (PUFAs), including linoleic acid (C18:2n6c), α-linolenic acid (ALA; C18:3n6) (at low levels) and eicosatrienoic acid (ETE; C20:3n3). With the exception of ETE, which is present in very low amounts in HM, omega-3 and omega-6 PUFAs are essential dietary components for infant health [26]. Univariate analysis revealed a statistically significant decrease in the total FA content in the pasteurized HM samples compared to the raw HM samples (* indicates a statistical significance of *p* ≤ 0.05), without exception (Figure 4).

#### 3.2.2. Heatmap Analysis

**Figure 5:**
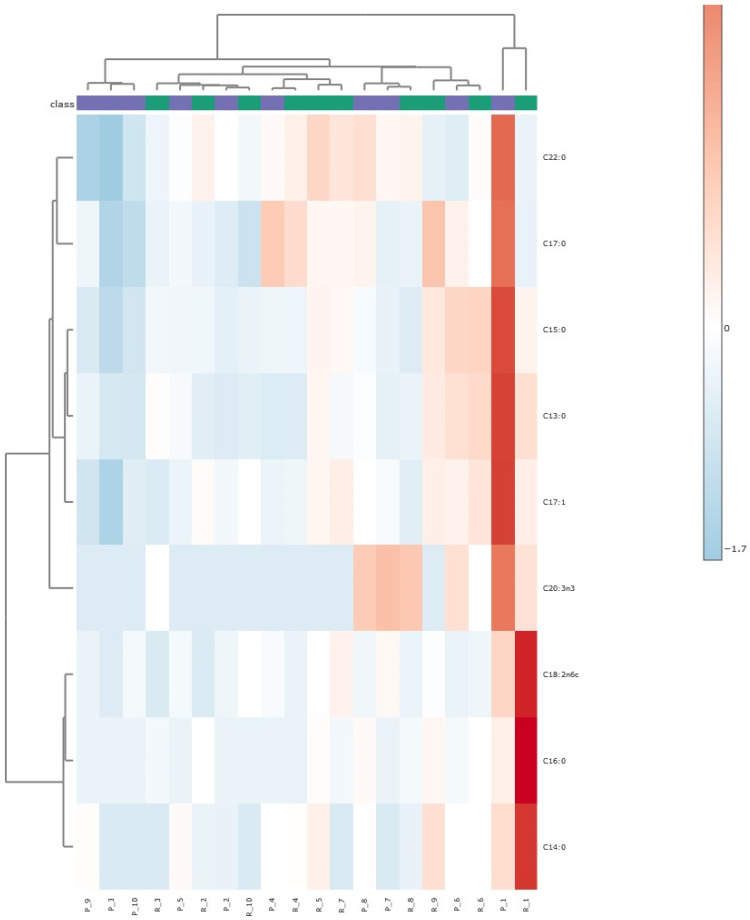
Heatmap representing the fatty acid (FA) abundance of pasteurized (P) and raw (R) milk samples. Clustering dendrogram of FAs (left) compared to individual samples (top dendrogram, purple represents pasteurized and green represents raw samples). The clustering of FAs (left axis) aids in identifying which lipids are co-regulated and respond similarly to the heat treatments. The color gradient, ranging from blue to red, indicates the relative abundance from low to high, respectively, for each lipid in each sample. In this analysis, both samples and FAs are hierarchically clustered, with dendrograms positioned at the top and left of the heatmap, indicating the clustering of samples and FAs, respectively.

A heatmap with the abundance of FAs across different samples, distinguished by individual sources and heat treatment conditions (“P” for pasteurized and “R” for raw), is shown in Figure 5. The clustering of samples according to heat treatment is represented by the top dendrogram, in which pasteurized samples are color-coded purple, and raw samples are colored green (shown below the dendrogram). The top dendrogram is separated into two high-level clusters: one cluster represents a single individual (both pasteurized and raw samples), and the other represents the remaining individuals. Within the second cluster, the observed dendrogram patterns suggest a strong effect of heat treatment on FA profiles, as samples from the same treatment condition clustered together (left part of dendrogram). Significant differences in FA profiles were seen between the two treatment conditions (raw or pasteurized) (Figure 5).

Overall, while individual variances do exist, pasteurization has had a stronger and more consistent effect on the FA profiles of HM samples than individual differences. This is evidenced by the distinct clustering of samples based on treatment conditions and the significant reductions in FA concentrations following HoP. It should be noted that Cruz et al., using the same procedure as we did, quantified FAs in HM ranging in size from 10:0 to 22:6 n-3 at higher concentrations [14]. It is likely that, in our hands, the heating to form fatty acid methyl esters (FAMEs) (FA derivatization step) resulted in the significant loss of short-chain and possibly some medium-chain FAs. The unreliability of this step has been noted [27], so, to expand the analysis, the volatile organic compounds (VOCs) were assessed by SPME and GC-MS. Although only semi-quantitative, this method captures a wider range of compounds.

#### 3.2.3. Volatile Organic Compounds Analysis by SPME and GC-MS

A summary of identified compounds, their chemical classes, their possible sources, and their physiological effects is presented in Table 1, together with retention time values in the Supplementary Data, Table S1. Of the 69 volatile organic compounds (VOCs) that were identified in HM, 50 were common to both raw and pasteurized milk, including various alcohols, aldehydes, alkanes, amines, aromatic compounds, ketones, terpenes, SCFAs, organochlorine compounds, and others. Medium-chain fatty acids (MCFAs) were also identified. Some VOCs were detected only in the pasteurized samples, notably furans and alkynes. Some compounds may have been lost due to volatilization during handling [28], although samples were kept at room temperature for minimum periods. Also, it is possible that some low-volatility VOCs and SCFAs may not have been captured.

**Figure 6:**
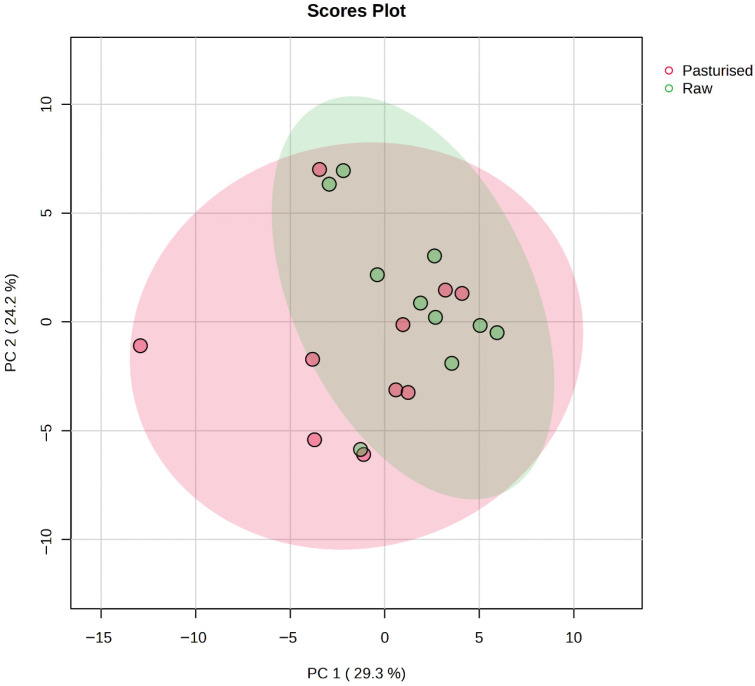
PCA plot of volatile organic compound profiles in raw and pasteurized human milk samples.

**Figure 7:**
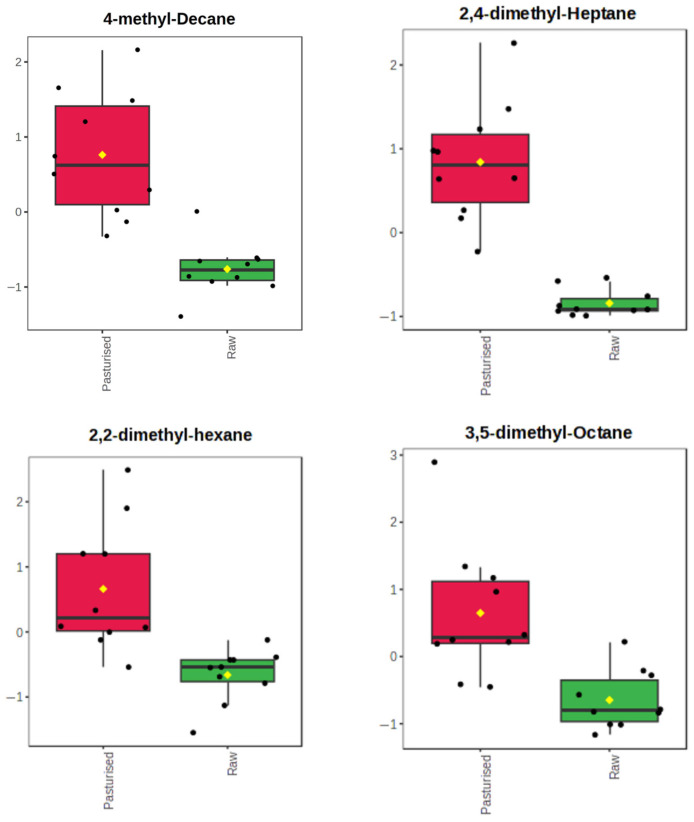
Boxplots comparing the fold-change in relative abundance of four volatile compounds (4-methyl-decane, 2,4-dimethyl-heptane, 2,2-dimethyl-hexane, and 3,5-dimethyl-octane) in pasteurized (red) and raw (green) milk samples. Black dots represent individual data points, and yellow diamonds represent the group mean.

A PCA plot (Figure 6) did not show a clear separation of data between the raw and pasteurized groups, although some degree of separation was apparent. This might be expected given the likely large variation in mothers’ diets and lifestyles, as well as their genetic makeup. The confidence interval of groups, represented by the ellipses (95% confidence interval), indicates greater variability for the pasteurized group compared to the raw group. Univariate analysis (Figure 7), including t-tests, revealed significant changes in the relative abundance of four alkanes, namely 4-methyl-decane, 2,2-dimethyl-hexane, 2,4-dimethyl-heptane, and 3,5-dimethyl-octane. Interestingly, all four compounds were higher in the pasteurized group compared to the raw group. Among them, 2,4-dimethyl-heptane was present at twice the concentration in pasteurized samples compared to raw.

**Figure 8:**
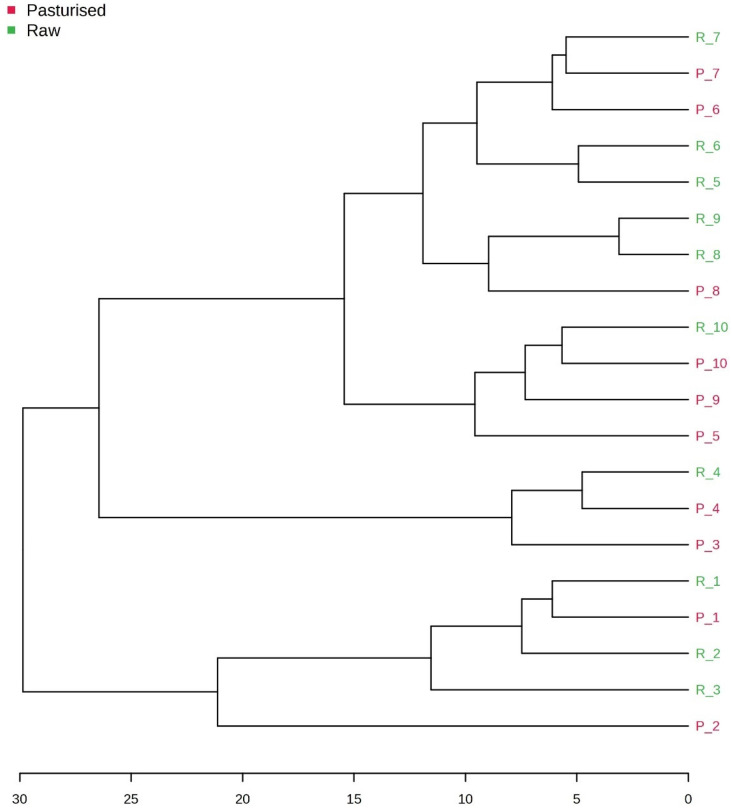
Hierarchical clustering dendrogram of pasteurized (P) and raw (R) milk samples’ volatile organic compounds (VOC) profile.

A dendrogram constructed by unsupervised clustering analysis (Figure 8) indicates that some data groups clustered closely together by treatment type (pasteurized or raw), while others clustered less closely. This indicates that the relative effect of heat treatment on HM varied between donors; for example, for four of the donors (1, 4, 7 and 10), the relative effect of heating on the VOC profiles was less pronounced than for the remainder, especially for donor 3, which showed deep branching (R3 vs. P3). Of course, individual genetic factors or other factors influencing the VOC profiles would also affect these results.

## 4. Discussion

Both FT-IR and GC analysis of HM samples in this study show that HoP treatment affects the HM lipidome and VOCs. FT-IR identified spectral features related to the functional groups of lipids, proteins, and carbohydrates, as well as their interactions within the milk matrix, revealing that HoP significantly affects the functional groups of lipids and proteins, as well as the bonds involved in lipid–protein and lipid–interactions. This was confirmed by second derivative analyses of the IR spectra (Figure 3). Changes in absorbance related to lipid and protein interactions were observed in the methylene (CH_2_) and carbonyl (CO) radicals at wavebands 2923 cm^−1^ and 2853 cm^−1^, respectively. These CH_2_ and CO functional groups were impacted by HoP, with an increase in absorbance in pasteurized samples compared to raw. This increase may result from damage to the milk fat globule membrane (MFGM), exposing more acyl chains that can interact with κ-caseins and casein micelles, and leading to an increase in the size of fat globules and the coalescence of fat globules [45,46,47]. These changes in fat globules could affect the bioavailability and digestibility of HM [48]. Moreover, the HM lipidome is distinct from that of other animals and IF, since it contains a higher proportion of ether lipids [2].

Changes in band intensity were also observed in the infrared spectral band at 1743 cm^−1^, which reflects variations in the levels of triacylglycerols (TAGs) and free fatty acids (FFAs), and also shows slight structural fluctuations in CO functional groups related to lipid–protein interactions [49,50,51]. Notably, the absorbance at 1743 cm^−1^ was higher in the pasteurized samples, again indicating damage to the MFGM, showing increased interactions between lipids and caseins through CO groups and corroborating the previous results. This is confirmed by other studies [50]. More generally, during the thermal degradation of food lipids, numerous complex chemical reactions occur, including oxidation, isomerization, and hydrolysis [52]. These reactions not only impact the nutritional value of the lipids but can also potentially produce toxic compounds, such as oxylipins, and off flavors in HM [13]. Detailed lipidomic studies of HM including LC analysis will provide further insights into these heat-induced changes.

The fatty acid analysis of HM identified significant concentrations of long-chain FAs (≥13 carbons), including saturated FAs like myristic and palmitic acids, and unsaturated FAs such as linoleic and eicosatrienoic acids, in all samples. Following HoP, there was a notable reduction in total FA content, with concentrations of all identified FAs significantly decreasing (*p* ≤ 0.05) (Figure 4). The decrease in long-chain FA concentrations supports the notion that HoP negatively affects lipid integrity. Further, heat treatment significantly impacted linoleic acid (an omega-6 PUFA) and ETE (an omega-3 PUFA), which is concerning as reductions in the former could have possible implications for infant growth and health. In general, PUFAs are involved in energy production, cell membrane structure, central nervous system development, and visual acuity [26,53]. Docosahexaenoic acid (DHA; 22:6n3) is a crucial PUFA needed for infant visual and neural development, especially in pre-term infants [54]. However, DHA was not detected in our study. One explanation may be the loss of PUFAs during sample derivatization, since, in the method used, samples were treated at 100 °C [14]. In comparison, saturated FAs are relatively more stable under these conditions. Notwithstanding this, we saw no unusual peaks in chromatograms indicating PUFA breakdown products. Although we cannot draw any conclusions regarding the effects of HoP on DHA, significant reductions in its levels would be of major clinical significance. The increase in methylene and ester groups observed in the infrared spectra also indicates lipid oxidation, which may contribute to the formation of rancid compounds affecting the taste and energy content of HM (or indeed any food) [52], so one would expect a negative impact from HoP in this regard.

Any loss of LCFAs in HM is particularly relevant for premature infants, as they are the primary recipients of DHM. Preterm infants have increased nutritional and energy requirements to compensate for reduced in utero growth and to support optimal postnatal development. The reduction in LCFA levels observed following HoP therefore underscores the importance of exploring alternative pathogen-reduction technologies that may better preserve the lipid fraction of HM, such as high-pressure processing [55]. However, the potential benefits of such approaches must be carefully balanced against possible trade-offs affecting other bioactive components of HM.

Heatmap and PCA analyses (Figure 5 and Figure 6, respectively) revealed separation between raw and pasteurized samples based on FA composition, with pasteurization having a stronger and more consistent effect on FA profiles than individual variability. Clustering analysis showed the co-clustering of certain FAs, such as C18:2n6c, C14:0, and C16:0, due to their similar abundance patterns, while others, like C15:0, C13:0, and C17:1 formed distinct clusters with correlated concentrations (Figure 5). The top clustering dendrogram in the heatmap further confirmed that data for samples that underwent HoP clustered together, as did the raw milk samples, with each group having its own more or less well-defined pattern. For most subjects, heat treatment showed a consistent effect on FA profiles whereby the effects of individual variation between samples were less than the effect of pasteurization.

To further extend our understanding of the changes in HM resulting from HoP, VOCs were analyzed in addition to the FAs. A total of 69 volatile organic compounds (VOCs) were identified in raw and pasteurized milks, with 50 compounds common to both and 18 found only in the pasteurized HM samples (Table 1). By comparison, using SPME and GC-MS, Muelbert et al. [56] identified 40 VOCs in breast milk samples collected from 170 mothers of pre-term infants, while He et al. [57] detected 128 VOCs in breastmilk samples from 60 mothers and 57 VOCs in six IFs, showing a similar level of VOC diversity. In our study, β-pinene was exclusive to raw milk, while other VOCs, like isooctane and furans, were unique to the pasteurized samples.

VOCs common between raw and pasteurized samples included alkanes, aldehydes, amines, ketones, a small number of halogenated VOCs and aromatics. These classes of VOCs are similar to those described by Gao et al. [58] and by Muelbert et al. [56] in relation to HM odor or flavor. Many of these VOCs identified in both milk types have been linked to maternal diet or metabolism, with terpenes and alcohols providing a more complex taste and aroma, as evidenced by comparing the VOC profiles of HM and IFs [29,57]. Terpenes have important flavor implications for infant feeding [56,58], and foods such as garlic and onion or dietary essential oils can affect the milk’s VOC profile. Studies have shown that terpenes, including β-caryophyllene, aromadendrene, and limonene, can be selectively transferred into HM, influencing its composition [59]. Our results confirmed the presence of caryophyllene in HM, a secondary metabolite of many natural products, and a known dietary cannabinoid that binds to CB2 non-psychoactive endocannabinoid receptors and has been shown to exert anti-inflammatory effects in mice [60]. Other terpenes detected in both the raw and pasteurized samples included the highly aromatic volatiles ylangene, humulene, limonene, and geranyl acetate. In contrast, β-pinene (also a terpene) was detected only in raw HM samples. This compound exhibits a wide range of pharmacological activities, including antibiotic resistance modulation, anticoagulant, antitumor, antimicrobial, antimalarial, antioxidant, anti-inflammatory, and analgesic effects [42].

Surprisingly, alkanes were detected at higher concentrations in the pasteurized milks compared to the raw samples. This was seen in the chromatograms and confirmed by fold-change analysis. Univariate analysis (Figure 7) similarly revealed that pasteurized HM had significantly higher concentrations of four alkanes, including 2,4-dimethyl-heptane, which was present at twice the concentration compared to raw HM. Moreover, 2,2-dimethyl-hexane, an isomer of isooctane, and 4-methyl-decane were observed only in the pasteurized group. Aromatic compounds and ketones were also detected at higher levels in the pasteurized samples, including isomers of xylene and 1,2,4-trimethylbenzene. Specific furans and alkynes were also found exclusively in the pasteurized milk samples. One such compound was 2-pentylfuran, characterized by a heterocyclic benzene ring with oxygen, commonly found in heat-processed foods and used as a food additive [44]. Interestingly, furans are also more prevalent in IFs which are heat-processed than in HM [32].

Of the 18 compounds detected in pasteurized HM only, it is possible that some, e.g., isomers of isooctane (C_8_H_18_), may have derived directly from the plastic containers, or via the mother through plastic exposure in the environment or from food [25,61,62]. Notwithstanding this, the possibility that some of these compounds may have originated from the polypropylene tubes used to heat-treat the samples cannot be fully discounted. Many (perhaps thousands) plastic-derived compounds have unknown health implications in food [25], and the implications of their introduction into HM are even less understood.

The presence of VOCs in HM originating from the diet, such as terpenes and other compounds, suggests a direct link between the mother’s diet and the taste and/or odor experience of babies fed HM, although some of these may also result from the oxidation of lipids [29]. This connection emphasizes the importance of dietary choices during lactation for optimal infant health outcomes. Notwithstanding thus, the possibility that some of these may have originated from the polypropylene tubes used to heat-treat the samples cannot be discounted. The impact of HoP affecting VOCs and the taste acceptance of DHM by babies is an interesting question [56,58].

An important consideration when analyzing VOCs is post-lactation factors related to milk bank processing that can change the profile of VOCs. Studies have shown that the pasteurization and storage of HM, as well as the type of feeding equipment used (e.g., plastic utensils), can significantly impact the composition of VOCs. For instance, one study found that VOCs such as cyclohexanone and 3,3,5-trimethylcyclohexanone can migrate from plastic-based feeding equipment used in neonatal intensive care units into HM [63]. The identification of exogenous compounds that could generate off-flavors and other potential toxic effects in pasteurized HM highlights the necessity of selecting appropriate equipment materials for milk processing, as well as investigating alternative methods to heat treatment (some of these methods are now being investigated [64]) that could have a lesser impact on milk’s VOC composition. A better understanding of the effects of heat treatment on the HM lipidome and VOC profiles, using a variety of omics techniques with a larger sample set, is also required.

## 5. Conclusions

This study demonstrated that HoP induced significant molecular alterations in the VOC and lipid profile of HM. FT-IR analysis proved a valuable technique, revealing marked changes in functional groups associated with lipid–protein interactions, suggesting the potential coalescence of milk fat globules and modifications to protein structures. Moreover, HoP led to an increase in lipid oxidation products, such as methyl and carbonyl species. These changes, together with the observed reduction in medium- and long-chain FAs highlight the potential detrimental impact of HoP on lipid integrity. Moreover, such changes, particularly FA oxidation products, would have significant impacts on olfactory cues for infant feeding. In addition to structural modifications, pasteurization clearly influenced the volatile profile of human milk. Variations in VOCs associated with maternal diet were detected, alongside possible exogenous compounds originating from plastic feeding equipment. The potential adsorption or loss of beneficial VOCs during treatment underscores the importance of selecting appropriate materials and processing methods to maintain the sensory and bioactive quality of DHM. Collectively, this study provides novel insight by identifying specific lipid oxidation markers, structural alterations, and VOC changes, parameters that remain largely unexplored in the literature on alternative pathogen-reduction technologies. Future research should focus on optimizing pasteurization conditions and exploring alternative processing technologies that better retain essential FAs, protein integrity, and bioactive volatile compounds, while maintaining microbiological safety, thereby ensuring the best possible nutrition for vulnerable newborns.

## Figures and Tables

**Table 1:** Volatile organic compounds (VOCs) identified in human milk (HM) in this study.

Samples	Class	Compound	Molecular Formula	Likely Origin/Pathways/Uses	Flavor/Other Possible Effects in HM	References
Raw and pasteurized HM	Alcohol	2-Ethylhexan-1-ol	C_8_H_18_O	Plant metabolite found in *Camellia sinensis*, *Alpinia chinensis*, and fruits. Used as a flavoring agent. Considered an indoor air pollutant indicator.	Linked to fetal developmental abnormalities.	[22,29]
		1-Octen-3-ol	C_8_H_16_O	Plant, animal and fungal metabolite. Used as a food additive. Found in mushrooms, *Allium* spp. and some herbs/spices.	Mushroom taste.	[23]
		1-Pentanol	C_5_H_12_O	Primary alcohol present in many food sources.	Strong smell and sharp taste.	[24]
		Geraniol	C_10_H_18_O	Found in many food sources of plant origin. Used as food additive and insect repellant.	Sweet, citrus, and floral odor/taste.	[23,29]
		Geranyl acetate	C_12_H_20_O_2_	Terpenoid constituent of various essential oils. Prepared as an ester.	Lavender odor.	[23,29]
		Arachidonic acid, methyl ester	C_21_H_34_O_2_	Human blood serum metabolite.		[24,29]
	Aldehyde	Gentisaldehyde (2,5 dihydroxybenzaldehyde)	C_7_H_6_O_3_	Found in all living organisms as a primary metabolite. Detected in foods of animal origin, such as poultry, bovine and porcine meat.	Antimicrobial properties against *Staphylococcus aureus*.	[29,30,31]
		2-Heptenal	C_7_H_12_O	In plant-based food sources, such as cereals and berries. Used as a food additive.	Pungent almond/fatty acid taste. Toxic effects at high concentrations.	[29]
		2-Nonenal	C_9_H_16_O	Plant metabolite and dietary compound.	Aroma component of beer and buckwheat.	[23,29]
		2-Octenal, (E)-	C_8_H_14_O	Found in Hops (*Humulus lupulus*) and in other organisms. Volatile oil component, used as food flavoring agent and fragrance.	Antifungal properties.	[23,29]
		5-Ethylcyclopent-1-enecarboxaldehyde	C_8_H_12_O	No sources identified		
		Pentanal	C_5_H_10_O	Plant metabolite, used in flavorings	Fermented, bready, fruity, nutty, berry odor	[23,29]
		Hexanal	C_6_H_12_O	Breakdown product of linoleic acid, present in animal and plant metabolomes	Apple, fresh, green, leafy and fruity aroma. In excess, it causes a “cardboard-like” off-flavor	[29,32]
		Heptanal	C_7_H_14_O	Part of the metabolism of all eukaryotes, also present in essential oils, dietary sources.	Citrus and fruity aroma, as well as green and herbal.	[24,29]
		Neral (β-citral)	C_10_H_16_O	Plant metabolite, component of essential oils in citrus and other fruits	Lemon aroma. Anti-inflammatory properties.	[23,29,33]
		Octanal	C_8_H_16_O	Found in fruits, herbs, and spices	Fruity, citrus and fatty taste	[29,32]
	Alkane	n-Hexane	C_6_H_14_	Organic solvent	Petroleum-like odor	[29]
		Decane	C_10_H_22_	Industrial substrate for petroleum products, rubber and paper	No expected adverse effects at low levels	[29,34]
		Dodecane	C_12_H_26_	Plant metabolite		[23]
		2,2,4,6,6-pentamethyl-heptane	C_12_H_26_	No sources identified		
		2,3,4-trimethyl-heptane	C_10_H_22_	No sources identified		
		2,4-dimethylheptane	C_9_H_20_	Fungal and plant metabolite		[23,29]
		3,3-dimethyl- heptane	C_9_H_20_	No sources identified	Acute exposure has neurotoxic effects	[29]
		2,3,3-trimethyl-pentane	C_8_H_18_	Human, mammalian and bacterial metabolite; overexpressed in bacterial strains exposed to pesticides		[24,29,35]
		4-methyl-decane	C_11_H_24_	A natural product found in foods such as cereals, pulses, and nuts. Also found in Poaceae (grasses) and Fabaceae (legumes).		[23]
		3,5-dimethyl-octane	C_10_H_22_	Metabolite detected in lung cancer metabolism		[29]
		4-methyl-octane	C_9_H_20_	Natural product found in tobacco (*Nicotiana tabacum*)		[29]
	Amine	(2-Aziridinylethyl)-amine	C_4_H_10_N_2_	No sources identified		
	Aromatic	1,3-Di-tert-butylbenzene	C_14_H_22_	Petroleum product used in polymer packaging		[25,29]
		Styrene	C_8_H_8_	Mainly used in the production of polystyrene plastics and resins.	Sweet, balsamic and floral tasting. Known carcinogen.	[29,36]
		Toluene	C_7_H_8_	Present in all living organisms, can be found in some foods, such as coriander. Used industrially as a feedstock and solvent. Traces also found in plastics that come into contact with food.	Paint-like taste. Significant toxicity and carcinogenicity associated with exposure.	[25,37]
	Fatty acid ester	n-Caproic acid vinyl ester (vinyl hexanoate)	C_8_H_14_O_2_	Plant metabolite		[23,29]
	Ketone	Acetone	C_3_H_6_O	Product of normal human metabolic processes and from environmental sources; used as an organic solvent in industry	Sensory irritant, toxic at high levels	[29]
		1-Hepten-3-one	C_7_H_12_O	An enone class of ketone compounds. It is a secondary metabolite, described in lacquered mushroom (*Ganoderma lucidum*) used in traditional Chinese medicine.		[38]
		6-methyl-2-heptanone	C_8_H_16_O	Metabolite found in *Aloe africana*, *Ceratophyllum demersum* and other organisms.		[22,23,29]
	Organochlorine	Chloroform	CHCl_3_	A non-polar solvent, used in industry.	Carcinogenic; central nervous system sedative effect	[29]
	Saturated fatty acid	Butanoic acid	C_4_H_8_O_2_	A human metabolite; also used as a food additive.	“Rancid,” “baby vomit,” or “strong cheese” aroma/odor. Oxidation products are key olfactory cues in infants.	[22,29,32]
		Caproic (hexanoic) acid	C_6_H_12_O_2_	Found in many animal fats and oils, human metabolite. Contributes to the flavor of vanilla and cheese. Constitutes significant proportion of goat’s milk fat.	Strong fatty, cheesy odor.	[22,29]
		Capric (n-Decanoic) acid	C_10_H_20_O_2_	Human, plant and algal metabolite. A constituent of goat’s milk.	Antibacterial agent and anti-inflammatory properties.	[22,29]
		Nonanoic acid	C_9_H_18_O_2_	Plant and algal metabolite.	Antifungal, plant antifeedant properties.	[22,23,29]
		Caprylic (octanoic) acid	C_8_H_16_O_2_	Naturally found in the milk of different mammals, as well as in palm and coconut oil	Fatty taste and odor	[23,29,32]
	Terpene	Geranial (α-Citral)	C_10_H_16_O	Acyclic monoterpenoid of plant origin. Present in many plant products, such as fruits, nuts, spices and herbs.	Bitter taste. Citrus and mint odor. Some reports of anti-inflammatory activity and trypanocidal properties.	[22,29]
		β-Caryophyllene	C_15_H_24_	Ubiquitous plant metabolite found in many taxa, including *Camellia sinensis* (green tea), *Piper nigrum* (black pepper), *Cinnamomum verum* (cinnamon) and *Cannabis sativa*.	Calming and sedative effects. Binds to endocannabinoid CB2 receptors. Gastric and colitis protective properties and anti-inflammatory.	[22,23,29,39]
		Caryophyllene oxide	C_15_H_24_O	Oxidized form of caryophyllene, found in various plant taxa. Used as a food additive and flavoring agent.	Does not have binding affinity to CB2 receptors but is described to have anticancer and analgesic properties.	[23,29,40]
		D-Limonene	C_10_H_16_	Major constituent of citrus oils (orange, lemon, lime, mandarin, etc.). Used as a flavor and fragrance additive.	Has antioxidant, anti-inflammatory, and immunomodulatory properties	[23,29,41]
		Humulene (α-Caryophyllene)	C_15_H_24_	A characteristic terpene of hops (*Humulus lupulus*), it is an open ring isomer of β-caryophyllene.	Hop odor and taste. Anti-inflammatory, cytotoxicity against cancer cells, antimalarial and antituberculosis activities.	[23,24,29]
		γ-Muurolene	C_15_H_24_	A sesquiterpene plant metabolite found in *Humulus lupulus*, basil, oats (*Avena sativa*) and other foods.		[23,29]
Raw HM only	Terpene	β-Pinene	C_10_H_16_	Isomer of pinene, is a ubiquitous plant metabolite found in various essential oils.	Woody, pine-like smell. Several pharmacological properties, including anti-inflammatory and anxiolytic	[29,42]
Pasteurized HM only	Aldehyde	Benzaldehyde	C_7_H_6_O	Present in natural sources, such as cherry and peach pits. Used as a flavoring agent.	Bitter and almond odor. Agonist of odorant receptors.	[23,29,32]
		Nonanal	C_9_H_18_O	A plant metabolite observed in essential oils. Human metabolite, found in cancer metabolism. Used in perfume making		[23,29,32]
	Alkane	2-methylheptane	C_8_H_18_	Octane isomer	Exposure to this and other petroleum compounds causes toxic effects; CNS depression, heart arrhythmia and pulmonary damage.	[29]
		4-methylheptane	C_8_H_18_	Octane isomer, plant metabolite		[29]
		2,2-dimethyl-hexane	C_8_H_18_	Octane isomer		[29]
		6-methyl-octadecane	C_19_H_40_	No sources identified		
		4,6-dimethyldodecane	C_14_H_30_	Human metabolite observed in ovarian cancer cell lines and head-and-neck cancer patients.		[29,43]
		Isooctane (2,2,4-trimethyl-pentane)	C_8_H_18_	Octane isomer used in petroleum industry	Toxic effects	[29]
		5-methylundecane	C_12_H_26_	Potential migrating and extractable food contact chemical		[25]
	Alkyne	1-Decyne	C_10_H_18_	Primary or secondary human metabolite		[22,29]
	Amine	2-aminopropan-1-ol	C_3_H_9_NO	Primary metabolite found in many living organisms	Fishy tasting. Physiological properties not described.	[24,29]
	Aromatic	1,2,4-Trimethylbenzene	C_9_H_12_	Used as a gasoline additive, in dyes and pharmaceuticals.	Acute levels cause headaches, nausea, vomiting and dizziness.	[29]
		o-Xylene	C_8_H_10_	Plant metabolite, also found in petroleum, coal, and tar.	Acute levels irritate the eyes, nose, throat and skin; causes headaches, dizziness, confusion, loss of muscle coordination	[23,29]
		p-Xylene	C_8_H_10_	Isomer of o-Xylene	See o-xylene	
	Furan	2-pentylfuran	C_9_H_14_O	Found in various heat-processed foods and drinks. Produced by oxidation of fatty acids, the Maillard reaction and the decomposition of carbohydrates.	Cooked, almond, burnt taste.	[22,29,44]
	Ketone	1-Octen-3-one	C_8_H_14_O	Flavoring agent.	Fresh mushroom or earthy metallic scent.	[29]
		2-Butanone	C_4_H_8_O	Bacterial metabolite in low levels. Used industrially as polar solvent	Sharp, sweet odor similar to acetone.	[29]
	Organochlorine	Dichloromethane	CH_2_Cl_2_	Used as solvent, degreaser and refrigerant.	Carcinogenic	[29]

## Data Availability

The original contributions presented in this study are included in the article/Supplementary Materials. Further inquiries can be directed to the corresponding author.

## Supplementary Materials

The following supporting information can be downloaded at: https://www.mdpi.com/article/10.3390/foods15071118/s1, Figure S1. Gas chromatograms comparing the fatty acid profiles of (A) raw human milk and (B) Holder pasteurized human milk. Table S1. Volatile organic compounds (VOCs) detected in human milk samples following different processing conditions, identified by GC–MS. Compounds are classified according to chemical class, with corresponding molecular formulas and retention times (minutes).
