# Supplementary material for: Holder Pasteurization Affects the VOCs and Lipid Profile of Human Milk
Source: Foods. 2026 Mar 24;15(7):1118. doi: 10.3390/foods15071118 (PMC13073039; doi:10.3390/foods15071118)
Supplement: Supplementary file 1 [file foods-15-01118-s001.zip › foods-4078161-supplementary.pdf]

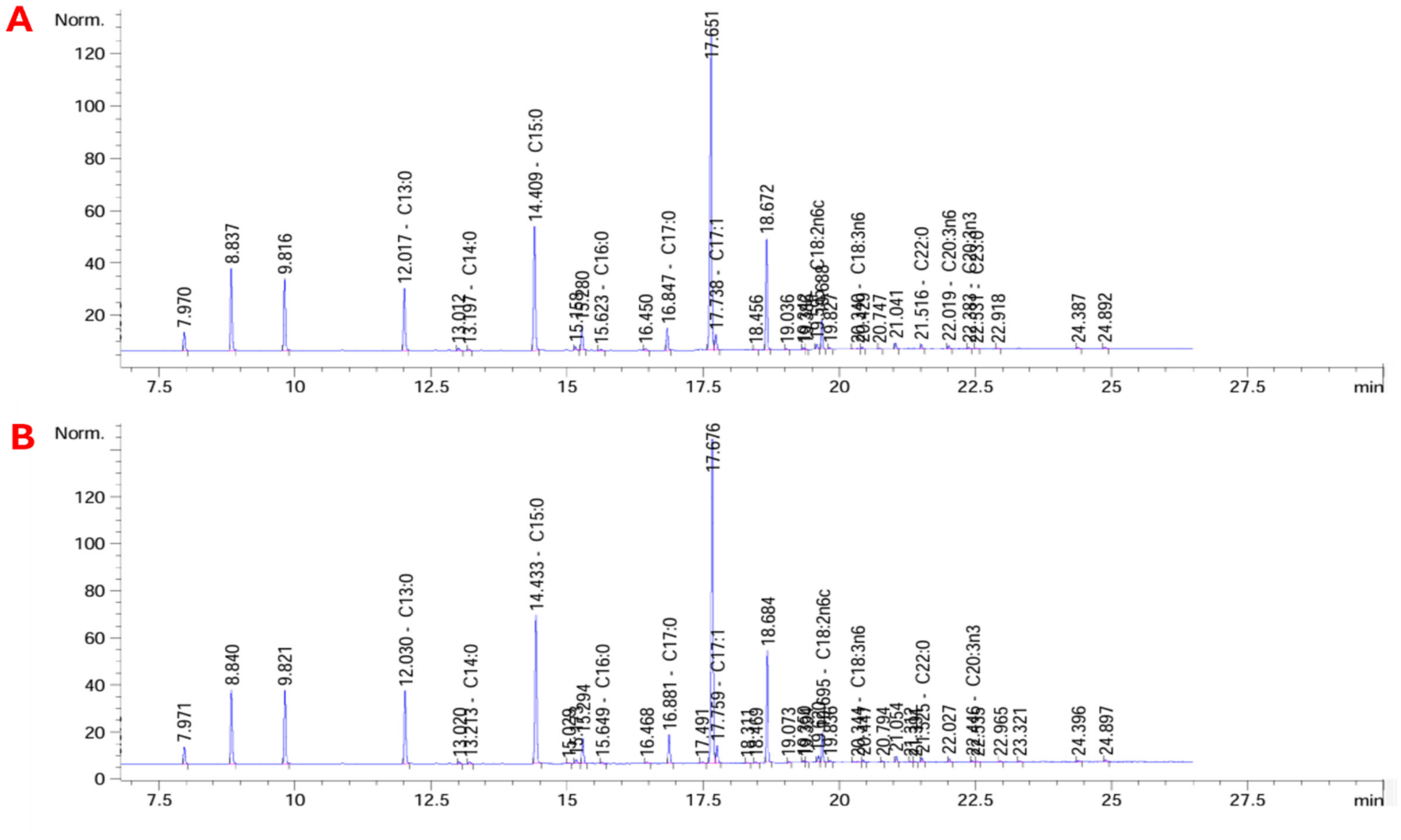

Figure S1. Gas chromatograms comparing the fatty acid profiles of (A) raw human milk and (B) Holder pasteurized human milk.

Table S1. Volatile organic compounds (VOCs) detected in human milk samples following different processing conditions, identified by GC–MS. Compounds are classified according to chemical class, with corresponding molecular formulas and retention times (minutes).

Class	Compound	Molecular Formula	Treatment (P, R or PR)	Retention time (minutes)
**Amine**	(2-Aziridinylethyl)amine	$C_4H_{10}N_2$	PR	1.496
**Alkane**	n-Hexane	C_6H_{14}	PR	1.513
**Alkane**	2-methyl-heptane	C_8H_{18}	P	1.834
**Alkane**	4-methyl-heptane	C_8H_{18}	P	1.861
**Alkane**	2,3,3-trimethyl-Pentane	C_8H_{18}	PR	1.867
**Alkane**	2,4-dimethyl-Heptane	C_9H_{20}	PR	2.033
**Ketone**	Acetone	C_3H_6O	PR	2.115
**Alkane**	4-methyl- Octane	C_9H_{20}	PR	2.314
**Ketone**	2-Butanone	C_4H_8O	P	2.676
**Organochlorine**	Methylene chloride (dichloromethane)	CH_2Cl_2	P	2.967
**Aldehyde**	Pentanal	$C_5H_{10}O$	PR	3.44
**Alkane**	Decane	$C_{10}H_{22}$	PR	3.566
**Alkane**	2,2-dimethyl-hexane	C_8H_{18}	P	3.633
**Alkane**	4-methyl-Decane	$C_{11}H_{24}$	PR	3.688
**Organochlorine**	Trichloromethane (chloroform)	$CHCl_3$	PR	3.93
**Alkane**	3,5-dimethyl-Octane	$C_{10}H_{22}$	PR	3.985
**Aromatic**	Toluene	C_7H_8	PR	4.16
**Alkane**	3,3-dimethyl-Heptane	C_9H_{20}	PR	4.238
**Aldehyde**	Hexanal	$C_6H_{12}O$	PR	4.67
**Terpene**	β-Pinene	$C_{10}H_{16}$	R	4.866
**Alkane**	5-methyl- undecane	$C_{12}H_{26}$	P	5.006
**Alkane**	2,3,4-trimethyl- Heptane	$C_{10}H_{22}$	PR	5.423
**Aromatic**	o-Xylene	C_8H_{10}	P	5.447
**Aromatic**	p-Xylene	C_8H_{10}	P	5.454
**Aldehyde**	Heptanal	$C_7H_{14}O$	PR	6.098
**Alkane**	Dodecane	$C_{12}H_{26}$	PR	6.107
**Terpene**	D-Limonene	$C_{10}H_{16}$	PR	6.199
**Alkyne**	1-Decyne	$C_{10}H_{18}$	P	6.666
**Furan**	2-pentylfuran	$C_9H_{14}O$	P	6.724
**Alkane**	4,6-dimethyl-dodecane	$C_{14}H_{30}$	P	6.767
**Alkane**	6-methyl-octadecane	$C_{19}H_{40}$	P	6.775
**Ketone**	6-methyl-2-Heptanone	$C_8H_{16}O$	PR	6.84
**Alcohol**	1-Pentanol	$C_5H_{12}O$	PR	7.012
**Aromatic**	Styrene	C_8H_8	PR	7.17
**Aromatic**	1,2,4-trimethyl-Benzene	C_9H_{12}	P	7.481
**Aldehyde**	Octanal	$C_8H_{16}O$	PR	7.548
**Ketone**	1-Hepten-3-one	$C_7H_{12}O$	PR	7.757
**Fatty acid ester**	n-Caproic acid vinyl ester	$C_8H_{14}O_2$	PR	8.064
**Aldehyde**	2-Heptenal, (Z)-	$C_7H_{12}O$	PR	8.091
**Aldehyde**	2-Heptenal, (E)-	$C_7H_{12}O$	PR	8.102
**Aldehyde**	Nonanal	$C_9H_{18}O$	P	9.01
**Aldehyde**	5-Ethylcyclopent-1-enecarboxaldehyde	$C_8H_{12}O$	PR	9.387

**Aromatic**	1,3-bis(1,1-dimethylethyl)-benzene (1,3-Di-tert-butylbenzene)	$C_{14}H_{22}$	PR	9.43
**Aldehyde**	2-Octenal, (E)-	$C_8H_{14}O$	PR	9.529
**Alcohol**	1-Octen-3-ol	$C_8H_{16}O$	PR	9.72
**Alcohol**	2-ethyl-1-hexanol	$C_8H_{18}O$	PR	10.235
**Aldehyde**	Gentisaldehyde	$C_7H_6O_3$	PR	10.556
**Aldehyde**	Benzaldehyde	C_7H_6O	P	10.841
**Aldehyde**	2-Nonenal, (E)-	$C_9H_{16}O$	PR	10.898
**Alcohol**	Arachidonic acid methyl ester	$C_{21}H_{34}O_2$	PR	11.549
**Terpene**	Caryophyllene	$C_{15}H_{24}$	PR	11.66
**Saturated**	Butanoic acid	$C_4H_8O_2$	PR	12.062
**Terpene**	Humulene	$C_{15}H_{24}$	PR	12.55
**Terpene**	Geranial (α-Citral)	$C_{10}H_{16}O$	PR	12.68
**Terpene**	Neral (β-Citral)	$C_{10}H_{16}O$	PR	12.68
**Terpene**	Geranyl acetate	$C_{12}H_{20}O_2$	PR	13.476
**Terpene**	γ-Muurolene (Naphthalene, 1,2,3,4,4a,5,6,8a-octahydro-7-methyl-4-methylene-1-(1-methylethyl)-, (1α,4aβ,8aα)-)	$C_{15}H_{24}$	PR	13.572
**Alcohol**	Geraniol	$C_{10}H_{18}O$	PR	14.47
**Saturated**	Hexanoic acid	$C_6H_{12}O_2$	PR	14.51
**Terpene**	Caryophyllene oxide	$C_{15}H_{24}O$	PR	16.04
**Saturated**	Octanoic acid	$C_8H_{16}O_2$	PR	16.701
**Amine**	2-amino-1-Propanol	C_3H_9NO	P	17.17
**Saturated**	Nonanoic acid	$C_9H_{18}O_2$	PR	17.72
**Saturated**	n-Decanoic acid	$C_{10}H_{20}O_2$	PR	18.695
